# Sex differences in at-risk drinking and associated factors–a cross-sectional study of 8,616 community-dwelling adults 60 years and older: the Tromsø study, 2015-16

**DOI:** 10.1186/s12877-022-02842-w

**Published:** 2022-03-01

**Authors:** Line Tegner Stelander, Anne Høye, Jørgen G. Bramness, Rolf Wynn, Ole Kristian Grønli

**Affiliations:** 1 Division of Mental Health and Substance Abuse, University Hospital of North Norway, P.O. Box 6124, 9291 Tromsø, Norway; 2 Department of Clinical Medicine, Faculty of Health Sciences, UiT The Arctic University of Norway, Tromsø, Norway; 3 Norwegian Institute of Public Health, Oslo, Norway

**Keywords:** Alcohol consumption, At-risk drinking thresholds, Older adults, Elderly, Sex differences, Self-reported health status, Mental distress, Public health, Tromsø Study, Norway

## Abstract

**Background:**

Alcohol consumption among older adults is on the rise, which may be an increasing public health concern. The proportion of older adults who drink above defined low-risk drinking limits, associated characteristics and the sex distribution of at-risk drinking vary across countries. The aims of this study were to (i) estimate the prevalence of at-risk drinking among older adults in Norway, (ii) investigate factors associated with at-risk drinking, and (iii) examine sex differences in alcohol consumption in the context of sociodemographic and selected health characteristics.

**Method:**

A cross-sectional study based on Tromsø 7 (2015–16), an ongoing population-based cohort survey. Data were retrieved from participants aged 60 and older (60-99 years) who answered questions about alcohol consumption (*n* = 8,616). Sex-stratified logistic regressions were used to assess the association between three at-risk drinking outcome variables, and sociodemographic and selected health characteristics. The outcome variables were operationalized using the Alcohol Use Disorders Identification Test (AUDIT), and Alcohol Consumption Questions (AUDIT-C), i.e. – cut off for at risk drinking, drinking any 6+ in the past year, and any alcohol problems.

**Results:**

The overall prevalence of at-risk drinking among those aged 60-99 years was equal in women and men; 44% and 46%, respectively. At-risk drinking was strongly associated with a higher level of education, with OR 2.65 (CI 2.28-3.10) in women and OR 1.73 (CI 1.48-2.04) in men.

**Conclusions:**

Almost half of older adults in Norway exceeded sex- and older adult-specific at-risk drinking thresholds. Our findings suggest some differences in factors associated with at-risk drinking between women and men. Explicitly, at-risk drinking was associated with very good health, living with a spouse or partner, and having adequate social support in women, while it was associated with the use of sleeping pills in men. Our findings suggest that women exceed at-risk drinking thresholds with better health, while men exceed at-risk drinking thresholds regardless of good or poor health.

**Supplementary Information:**

The online version contains supplementary material available at 10.1186/s12877-022-02842-w.

## Background

Alcohol use among older adults may be a public health concern, as evidence of increasing alcohol consumption among older adults is growing [1–6]. Alcohol use represents a major cause of injury and mortality and is causally linked to a high number of diseases that are common in older adults [7]. As aging occurs, lean body mass and total body water decrease, and thus the same level of alcohol intake results in higher levels of blood alcohol content in older adults than in younger adults [4]. Furthermore, the liver’s capacity to metabolize alcohol may be reduced, and biological changes to other internal organs and the brain (e.g., neuronal receptor sensitivity increase) can result in increased susceptibility to the harmful effects of alcohol [4, 8]. Older adults have higher rates of comorbidity and prescribed and over-the-counter drug use, and these factors may contribute to higher vulnerability to the detrimental effects of alcohol compared to younger adults [9].

In Europe, in 2016, the total alcohol per capita consumption among drinkers was 9.8 L, but 40% of the population (15+ years) had abstained from alcohol in the past 12-months [10]. In Norway, the total alcohol per capita consumption among drinkers was 9.4 L, and 21% had abstained from alcohol in the last year. Among adults aged 60 and older, the proportion who reported drinking alcohol at least twice weekly increased from 9-11% in 1994-95 to 25-35% in 2015-16, whereas the proportion among those aged 16-59 increased from 12 to 16%, which indicates a shift from younger to older regular drinkers of alcohol in Norway [11, 12]. There is growing evidence that “baby boomers” (those born between 1946 and 1964) are bringing their riskier drinking habits into old age [3, 5]. However, about one-third of older adults who develop drinking problems did not have drinking problems earlier in life, which may lead to this not being suspected as a problem by the physician [3]. Detection of harmful alcohol use in older adults can also be difficult, due to an atypical presentation (such as falls, incontinence, confusion, sleep problems, reduced or increased pharmacological effect of chronic therapies), or because it is masked by comorbid physical or psychiatric illness. [13].

Excessive drinking in later life, as opposed to low-risk drinking, has been associated with male sex, being closer to middle age, less than college education, poor physical health status, polypharmacy, cognitive impairment, poor mental health, loneliness, living alone (among men), size of social network, and social isolation [8, 14]. However, the factors listed here are based on studies performed in the US, since there is still a shortage of studies on alcohol use and associated characteristics among the current generation of older people in Europe [15].

The definition of “at-risk” drinking among older adults varies between studies [5, 16, 17]. The US National Institute on Alcohol and Alcoholism (NIAAA) advises that adults limit alcohol intake to 2 drinks or less in a day for men and 1 drink or less in a day for women, and those who take certain over-the-counter or prescription medications or have certain medical conditions should avoid alcohol completely [18]. However, there are currently no commonly accepted thresholds for at-risk drinking in older adults, and the use of different screening tools and populations ranging from community dwelling to psychiatric inpatients in the studies, has contributed to differing prevalence estimates of at-risk drinking, from approximately 10% in the US to 45% in New Zealand and the North European countries [2, 19–21]. There are several screening tools for at-risk alcohol use that have been validated in older adults [22]. One of these instruments, the Alcohol Use Disorders Identification Test (AUDIT), was developed by the World Health Organization as a method of screening for excessive drinking [23]. Both the full 10-item AUDIT, and the shorter three-item AUDIT-C, have been used in a variety of settings, including among community dwelling older adults, and have been shown to have a good ability to correctly identify those with unhealthy drinking habits [22].

Although some previous studies have investigated risky drinking patterns in older adults [14, 17, 19–21, 24–26], differentiated knowledge from various cultural settings is needed to identify the prevalence and predictive factors for at-risk drinking in this fast-growing segment of the population [27]. Furthermore, there is little research on sex differences related to the characteristics of at-risk alcohol consumers in the current generation of older adults. Filling this knowledge gap is important, to reduce the under detection and misdiagnosing of the health-related consequences of excessive alcohol use. The aims of this study were to (i) estimate the prevalence of three outcomes of at-risk drinking among older adults (defined as those aged 60 and older) in Norway, (i.e. AUDIT-C threshold of ≥3 for women and ≥4 for men, drinking any 6+ in the past year, and reporting any alcohol problems), (ii) investigate factors associated with at-risk drinking, and (iii) examine sex differences in alcohol consumption in the context of sociodemographic and selected health characteristics.

## Materials and methods

### Study design and study sample

This study is a cross-sectional examination of data from the Tromsø Study, an ongoing population-based cohort study conducted in the municipality of Tromsø, the seventh-largest city in Norway [28]. The present study is based on Tromsø 7 (2015–16) and is conducted to investigate factors associated with alcohol consumption in the current generation of older adults. Data were retrieved from participants aged 60 and older at the time of participation who answered questions about alcohol consumption. All residents of Tromsø municipality aged 40 and older were invited to participate in the survey. Eligible for this study were 8,616 out of 12,973 invited participants aged 60 to 99 years at the time of participation, which represented a participation rate of 66% (Table 1).

**Table 1:** Sample description: Participants ≥60 years (*n *= 8,616) in the Tromsø survey (2015-16)

Characteristics	Number	Valid percentage
Total (attendance, %)	8,616 (66.4)	100
Sex		
Female (attendance, %)	4,451 (65.4)	51.7
Male (attendance, %)	4,165 (67.6)	48.3
Age		
60-69	5,179	60.1
70–79	2,676	31.1
≥80	761	8.8
Education		
Elementary school (up to 10 years)	3,054	36.6
High school (up to an additional three years)	2,207	26.5
College/university (at least four additional years)	3,075	36.9
Missing	280	
Relationship status		
Living with a spouse/partner	5,905	72.9
Living alone	2,199	27.1
Missing	512	
Enough social support		
Yes	7,197	86.4
No	1,132	13.6
Missing	287	
Self-reported health status		
Excellent	885	10.4
Good	4,497	52.9
Neither good nor bad	2,652	31.2
Bad or very bad	468	5.5
Missing	114	
HSCL-10 (cut-off 1.85)		
Yes	456	5.7
No	7,481	94.3
Missing	679	
Have used sleeping pills during last four weeks		
Yes	1,034	13.0
No	6,950	87.0
Missing	632	

### Study variables

*Social and demographic variables.* Sex was stratified as women and men. Age was measured as a continuous variable and subsequently recoded into three age groups: 60–69 years, 70–79 years and 80 years and older (80-99). Educational level was categorized as (1) primary/elementary school, (2) secondary/upper secondary education (up to an additional three years) and (3) college/university/tertiary education (at least four additional years). Relationship status was assessed by the following question: “Do you live with a spouse/partner?” with the response alternatives: Yes or No. Social support/loneliness was measured by the following question: “Do you have enough friends you can talk confidentially with?” with the response alternatives: Yes or No.

*Health characteristics*. Self-reported health (SRH) is a subjective measure of the current state of health. SRH has been widely used in population surveys and is a well-known predictor of future health outcomes, use of health services and mortality in adults over 60 years, and is often used as a replacement instrument of comorbidities [29–31]. It was measured by the following question: “How do you in general consider your own health to be?” Response alternatives were categorized as (1) bad or very bad, (2) neither good nor bad, (3) good, and (4) excellent. Mental health was assessed using The Hopkins Symptom Check List-10 (HSCL-10), an abbreviated version of the original HSCL-90 [32]. This ten-item questionnaire is a widely used, self-administered instrument designed to measure mental distress (symptoms of depression and anxiety) in population surveys [33]. The suggested cut-off limit of HSCL-10 ≥1.85 [34] was used to dichotomize mental distress: Yes or No. One question about the use of sleeping pills during the last four weeks was included (not used, less frequently than every week, every week, but not daily, or daily), as the combination of z-hypnotics and alcohol consumption has been found to be high among older adults in Norway [35]. Response alternatives were dichotomized: Have used/Have not used sleeping pills during the last four weeks.

*Alcohol consumption* was measured by extracting the first three items of the 10-item Alcohol Use Disorders Identification Test (AUDIT) [23], often labelled AUDIT-C (“C” for consumption). AUDIT-C consists of questions on the frequency of drinking (0=never, 1=monthly or less, 2=2-4 times a month, 3=2-3 times a week, or 4=4 or more times a week), number of units on a typical drinking day (0=1-2, 1=3-4, 2=5-6, 3=7-9, or 4=10 or more), and frequency of heavy episodic drinking (HED) defined as ≥6 units (0=never, 1=less than monthly, 2=monthly, 3=weekly, 4=daily or almost daily). In Norway, one unit of alcohol is defined as 12 g of ethanol.

We used a threshold specific to older age to define at-risk drinking, suggested by Towers et al. [19], with a sex-specific AUDIT-C threshold of ≥3 for women and ≥4 for men. The third AUDIT-C item, often referred to as AUDIT-3, is recommended as an independent screen of risky alcohol use in primary health care by the NIAAA. We used an AUDIT-3 threshold of ≥1 (i.e., one or more instances of drinking ≥6 units/≥72 g of ethanol in one sitting during the past year), instead of ≥2 which is more often used (i.e., one or more instances of drinking ≥6 units/≥72 g of ethanol in one sitting during the past month), to identify older adults for whom any level of bingeing is strongly associated with adverse consequences [14].

*Problems related to alcohol use* were assessed with AUDIT items 4-10, often labelled AUDIT-P (“P” for problems). We examined those who scored ≥1 when summing the score on AUDIT items 4-10, thus reflecting any alcohol-related problem as done by others [36].

### Statistics

Continuous variables are presented as the mean (SD), and categorical variables are presented as counts (%). Chi-square tests were used to assess associations between drinking categories and sociodemographic and health characteristics.

We used logistic regression models to assess the association between the at-risk drinking outcome variables as binary responses and sociodemographic and health characteristics as independent variables. To examine whether the effect of the independent variables differed for men and women, we tested for interaction by including two-way cross product terms in the models. We observed significant interactions between at-risk drinking and most of the independent variables. Thus, all logistic regression analyses were stratified by sex (men and women). Each drinking category was analysed separately without creating mutually exclusive groups. At-risk drinkers were compared with low-risk drinkers, heavy episodic drinkers with non-heavy episodic drinkers, and participants experiencing some sort of alcohol problems with those not experiencing alcohol problems. Only participants responding affirmatively to having consumed alcohol during the last 12 months were included in these analyses. Due to the large sample size, listwise deletions for missing values were used. Three sets of logistic regression analyses were conducted to model various categories of at-risk drinking as a function of sociodemographic factors, perception of having enough social support, perception of general health, mental distress, and the use of sleeping pills. Associations between the dependent variables and sociodemographic characteristics and selected health variables were investigated, first in unadjusted models. Subsequently, we controlled for other variables by building multiple logistic regression models. Age and educational level were significantly associated with all drinking behaviours in both men and women and were included in the final models. The results are presented as odds ratios (ORs) with 95% confidence intervals (CIs). Levels of significance at both 0.05 and 0.01 are provided in the tables, but given the large sample size, the main findings at the 0.01 level are discussed in the article.

All analyses were conducted using IBM SPSS Statistics for Windows, version 27.

## Results

### Sample Characteristics

The mean age of the included older adults (*n *= 8,616) was 68.9 (SD 6.9) years, and 52% were women. Participants who had a higher level of education (>12 years) numbered 3,075 (37%), and 5,905 (73%) lived with a spouse. Overall, 5,382 (63%) of the participants reported good or excellent health status, 1,034 (13%) had used sleeping pills during the last four weeks, 456 (6%) were mentally distressed, and 1,132 (14%) experienced not having enough social support (Table 1).

### Drinking patterns

Overall, 639 (14%) women and 305 (7%) men reported not drinking during the past year (*p *< 0.01). The prevalence of men versus women exceeding the sex- and older adult-specific threshold for at-risk drinking was not significantly different: the results were 46% in men and 44% in women (*p *= 0.117). Among 60- to 69-year -olds, 50% of women and 54% of men were at-risk drinkers; among 70- to 79-year -olds, 36% of both women and men were at-risk drinkers; and among 80- to 99-year -olds, 24% of women and 19% of men were at-risk drinkers. In the categories of heavy episodic drinking (AUDIT-3 ≥1) and alcohol problems (AUDIT items 4-10 ≥1), significant sex differences were found, with a higher prevalence among men than among women (*p *< 0.001 for both categories) (see Table 2). Among current drinkers who reported heavy episodic drinking, 19% of women and 43% of men reported this less than monthly, 2.4% of women and 10.5% of men reported this monthly, 0.9% of women and 3.2% of men reported this weekly, and 0.1% of women and 0.3% of men reported this daily or almost daily.

**Table 2:** Prevalence of drinking patterns according to selected characteristics: Participants ≥60 years (*n *= 8,616) in the Tromsø survey (2015-16)

	Full sample (*n *= 8,616)		Different patterns of drinking, current drinkers (*n *= 7,672)		
Characteristics	Non-drinkers, n (%)	Low-risk drinking, n (%)	At-risk drinking^e^, n (%)	Heavy episodic drinking^f^, n (%)	Alcohol problems^g^, n (%)
Overall prevalence	944 (11.1)	4,173 (55.0)	3,409 (45.0)	3,004 (39.9)	1,644 (22.1)
Sex					
Female	639 (14.6)^a^	2,099 (55.9)^a^	1,653 (44.1)^a^	844 (22.7)^a^	446 (12.1)^a^
Male	305 (7.4)^a^	2,074 (54.2)^b^	1,756 (45.8)^b^	2,160 (56.8)^a^	1,198 (31.9)^a^
Age (years)					
60-69	368 (7.1)^a^	2,296 (48.0)^a^	2,484 (52.0)^a^	2,192 (46.1)^a^	1,245 (26.4)^a^
70–79	384 (14.5)^a^	1,449 (64.2)^a^	809 (35.8)^a^	720 (32.2)^a^	376 (17.1)^a^
≥80	192 (26.1)^a^	428 (78.7)^a^	116 (21.3)^a^	92 (17.3)^a^	23 (4.5)^a^
Educational level					
Elementary school	496 (16.4)^a^	1,661 (65.7)^a^	869 (34.3)^a^	923 (36.9)^a, b^	435 (17.7)^a^
High school	196 (8.9)^a^	1,098 (54.8)^a^	904 (45.2)^a^	801 (40.3)^b^	449 (22.7)^a^
College/university	193 (6.3)^a^	1,287 (44.8)^a^	1,587 (55.2)^a^	1,220 (42.6)^a^	731 (25.8)^a^
Relationship status					
Living alone	332 (15.2)^a^	1,095 (59.3)^a^	753 (40.7)^a^	653 (35.6)^a^	381 (21.3)^a^
Living with a spouse/partner	509 (8.7)^a^	2,843 (52.9)^a^	2.527 (47.1)^a^	2,227 (41.7)^a^	1,207 (22.8)^b^
Enough social support					
No	160 (14.2)^a^	561 (58.2)^a^	403 (41.8)^a^	405 (42.3)^a^	265 (28.3)^a^
Yes	733 (10.2)^a^	3,475 (54.1)^a^	2,950 (45.9)^a^	2,544 (39.9)^b^	1,347 (21.4)^a^
Self-reported health status					
Bad or very bad	103 (22.5)^a, b^	205 (57.7)^a^	150 (42.3)^a^	131 (37.6)^a^	98 (28.8)^a, b^
Neither good nor bad	382 (14.5)^a ,b^	1,342 (59.8)^b^	903 (40.2)^b^	862 (38.8)^b^	496 (22.7)^a^
Good	377 (8.4)^a^	2,206 (53.9)^a, b^	1,890 (46.1)^b^	1,649 (40.5)^c^	891 (22.1)^b^
Excellent	62 (7.0)^b^	373 (45.5)^a, b, c^	446 (54.5)^a, b, c^	340 (41.5)^d^	150 (18.4)^a, b^
Mental distress^h^					
No	761 (10.3)^a^	3,623 (54.5)^a^	3,026 (45.5)^a^	2,672 (40.5)^a^	1,434 (21.6)^a^
Yes	72 (16.0)^a^	186 (49.2)^a^	192 (50.8)^a^	147 (39.5)^b^	138 (36.6)^a^
Have used sleeping pills during last 4 weeks					
No	658 (9.5)^a^	3,397 (54.3)^a^	2,862 (45.7)^a^	2,548 (40.9)^a^	1,370 (22.3)^a^
Yes	171 (16.7)^a^	453 (53.2)^b^	399 (46.8)^b^	279 (33.2)^a^	196 (23.4)^b^

Pairs that share superscript letters within the same independent variable category and column are significantly different (a, b, c, d = *p *< 0.05).

^e^AUDIT-C ≥3 for women and AUDIT-C ≥4 for men suggest at-risk alcohol use; ^f^AUDIT-3 ≥1, ≥6 units (≥72 grams of pure ethanol) in one sitting at least once last 12 months; ^g^AUDIT items 4-10 ≥1; ^h^HSCL-10 cut-off ≥1.85

Significant bivariate associations between at-risk drinking and several of the determinants were also observed. At-risk drinking was more prevalent among persons of lower age, with higher educational levels, who were living with a spouse or partner, who had enough social support, who had better self-rated health status, and those who were mentally distressed.

### Factors associated with different patterns of at-risk drinking

The sex-stratified logistic regression modelling of at-risk drinking patterns confirmed but also differentiated some of the bivariate associations observed in the full sample. We observed significant interactions in at-risk drinking by sex and increasing age group (*p* = 0.017), increasing educational level (*p *< 0.001), living with a spouse or partner (*p *< 0.001), having enough social support (*p *= 0.045), and increasing SRH (*p *= 0.002). In the heavy episodic drinking category, significant interactions were observed by sex and increasing age group (*p *= 0.005) and living with a spouse or partner (*p *= 0.012). In the alcohol problem category, significant interaction according to sex and increasing educational level was observed (*p *= 0.021).

Lower age and higher educational level were positively associated with exceeding an AUDIT-C threshold of ≥3 for women (Table 3) and ≥4 for men (Table 4). Better SRH status was positively associated with at-risk drinking in women, while this association was not observed in men. Living with a partner was positively associated with at-risk drinking in women but not in men. Having used sleeping pills during the last four weeks was positively associated with at-risk drinking in men but not in women.

**Table 3:** Factors associated with different patterns of at-risk drinking: Women ≥60 years (current drinkers, *n *= 3,752) in the Tromsø survey (2015-16)

	At-risk drinking^a^ (*n *= 1,653) vs. low risk drinking (*n *= 2,099)		Heavy episodic drinking^b^ (*n *= 844) vs. no heavy episodic drinking (*n *= 2,871)		Alcohol problems^c^ (*n *= 446) vs. no alcohol problem (*n *= 3,228)	
Predictor	Adjusted OR﻿†	95% CI	Adjusted OR﻿†	95% CI	Adjusted OR﻿†	95% CI
Age (ref: 60-69)	1.00	ref	1.00	ref	1.00	ref
70–79	0.57**	0.50-0.67	0.51**	0.42-0.62	0.48**	0.37-0.62
≥80	0.30**	0.22-0.40	0.31**	0.21-0.48	0.03**	0.01-0.19
Educational level (ref group: Elementary school)	1.00	ref	1.00	ref	1.00	ref
High school	1.80**	1.52-2.12	0.85	0.69-1.05	1.18	0.90-1.55
College/university	2.65**	2.28-3.10	0.94	0.78-1.14	1.37*	1.07-1.75
Living with a spouse or partner (vs. living alone)	1.42**	1.22-1.64	0.95	0.80-1.14	0.71**	0.57-0.89
Enough social support (vs. not)	1.41**	1.12-1.77	0.98	0.74-1.30	0.70*	0.50-0.98
Self-reported health status (ref group: Bad or very bad)	1.00	ref	1.00	ref	1.00	ref
Neither good nor bad	1.40*	1.01-1.95	1.05	0.70-1.58	0.82	0.50-1.33
Good	1.66**	1.21-2.29	0.91	0.61-1.35	0.67	0.42-1.08
Excellent	2.33**	1.62-3.34	1.13	0.73-1.76	0.51*	0.30-0.88
Mental distress (vs. no mental distress)	0.92	0.71-1.18	1.02	0.75-1.39	2.83**	2.08-3.85
Have used sleeping pills during last 4 weeks (vs. no use last 4 weeks)	1.08	0.91-1.30	1.19	0.95-1.49	1.90**	1.46-2.48

*OR* Odds Ratio, *CI* Confidence Interval

^a^AUDIT-C ≥3, suggests at-risk alcohol use in women; ^b^AUDIT-3 ≥1, ≥6 units (≥72 g of pure ethanol) in one sitting at least once last 12 months; ^c^AUDIT items 4-10 ≥1

†Adjusted for age (continuous) and educational level

*p ≤ 0.05 **p < 0.01

**Table 4:** Factors associated with different patterns of at-risk drinking: Men ≥60 years (current drinkers, *n* = 3,830) in the Tromsø survey (2015-16)

	At-risk drinking^a^ (*n* = 1,756) vs. low risk drinking (*n *= 2,074)		Heavy episodic drinking^b^ (*n *= 2,160) vs. no heavy episodic drinking (*n *= 1,645)		Alcohol problems^c^ (*n *= 1,198) vs. no alcohol problem (*n *= 2,559)	
Predictor	Adjusted OR﻿†	95% CI	Adjusted OR﻿†	95% CI	Adjusted OR﻿†	95% CI
Age (ref group: 60-69)	1.00	ref	1.00	ref	1.00	ref
70–79	0.50**	0.43-0.57	0.46**	0.40-0.54	0.57**	0.49-0.67
≥80	0.22**	0.16-0.31	0.15**	0.11-0.20	0.17**	0.11-0.27
Educational level (ref group: Elementary school)	1.00	ref	1.00	ref	1.00	ref
High school	1.24*	1.04-1.48	0.95	0.79-1.13	1.11	0.92-1.34
College/university	1.73**	1.48-2.04	0.90	0.76-1.06	1.19	1.00-1.41
Living with a spouse or partner (vs. living alone)	1.00	0.84-1.19	0.80*	0.67-0.96	0.82*	0.68-0.98
Enough social support (vs. not)	1.10	0.92-1.32	1.18	0.98-1.42	0.79*	0.66-0.96
Self-reported health status (ref group: Bad or very bad)	1.00	ref	1.00	ref	1.00	ref
Neither good nor bad	0.82	0.59-1.13	1.25	0.90-1.74	0.78	0.56-1.09
Good	0.87	0.64-1.19	1.32	0.96-1.82	0.64**	0.46-0.88
Excellent	0.84	0.58-1.21	1.19	0.82-1.74	0.46**	0.31-0.68
Mental distress (vs. no mental distress)	1.24	0.85-1.80	1.35	0.90-2.00	2.29**	1.59-3.32
Have used sleeping pills during last 4 weeks (vs.no use last 4 weeks)	1.58**	1.22-2.05	1.18	0.90-1.54	1.57**	1.20-2.05

*OR *Odds Ratio, *CI *Confidence Interval

^a^AUDIT-C ≥4, suggests at-risk alcohol use in men; ^b^AUDIT-3 ≥1, ≥6 units (≥72 g of pure ethanol) in one sitting at least once last 12 months; ^c^AUDIT items 4-10 ≥1

†Adjusted for age (continuous) and educational level

*p ≤ 0.05 **p < 0.01

Some of the bivariate associations not yielding significance in the full sample yielded significant associations in sex-stratified logistic regression analyses, and vice versa. For example, in bivariate analyses, the use of sleeping pills was not associated with at-risk drinking, whereas sex-stratified modelling found that men, but not women, had a higher probability of at-risk drinking if they consumed sleeping pills. In contrast, some bivariate associations yielding significance in the full sample lost significance in sex-stratified models. For example, mental distress was associated with at-risk drinking in bivariate analyses. However, sex-stratified modelling showed results in the opposite direction for this correlation, and thus, this connection was offset. Sex-stratified modelling also showed that only women contributed to the association between better SRH status and at-risk drinking observed in the bivariate analyses. The same was true for the association between having enough social support and at-risk drinking.

Only increasing age was associated with a lower probability of heavy episodic drinking for both women and men. None of the other independent factors were associated with this drinking pattern in women, whereas living with a spouse or partner lowered the probability of heavy episodic drinking in men.

Increasing age, living with a spouse or partner, having enough social support, and better health reduced the probability of alcohol problems in both women and men. Mental distress and the use of sleeping pills were strongly associated with a greater likelihood of alcohol problems in both sexes. Educational level was not associated with any alcohol problems in men, whereas having a college or a university degree (at least an additional four years) was associated with a higher probability of alcohol problems in women.

## Discussion

The overall prevalence of at-risk drinking among those aged 60-99 was found to be as high as 44% and 46% in women and men, respectively, when utilizing sex- and older adult-specific thresholds. Subjects of younger age (60-69 years) reported higher alcohol consumption and more prevalent at-risk drinking. In both sexes, at-risk drinking was associated with a higher level of education, with a stronger correlation in women. Women with better health were more likely to report at-risk drinking. This was also true for women living with a spouse or partner, or when they reported having adequate social support. Men who reported using sleeping pills were more likely to exceed at-risk drinking thresholds. This association was not found in women.

The finding of an equal prevalence of at-risk drinking in older women and men reflects substantial evidence that sex differences in alcohol consumption are reduced, and even absent in some age groups [1, 2, 12, 37–39]. The high prevalence of at-risk drinking, even among the oldest old, challenges the assumption that increased drinking among older people is a result of the baby boomers` more risky drinking patterns, as participants in both oldest age groups (70-79 years and 80 years and older) belong to the cohorts preceding the baby boomers. Furthermore, our findings stand in contrast to previous literature, which has shown that women reduce at-risk drinking more than men in old age [2, 5, 38]. Norwegian older adults have greater financial security, better health and welfare systems, and less social inequality than in the US and many other European countries, which may increase the availability and possibility of higher alcohol consumption [12]. Additionally, there is a high degree of gender equality, as women have increased their work participation and have improved the socioeconomic status relative to men’s over the last decades [40]. Our findings may reflect a north-south gradient across European countries in values and attitudes towards gender equality, which results in more equal drinking between the sexes, as has been reported from other studies [12, 39, 40]. However, most older people in Norway report to drink frequently, but only a few alcohol units (one-two) on each occasion [11, 12]. This implies an AUDIT score ≥4, but may not involve risky drinking.

Both heavy episodic drinking and experiencing some sort of alcohol problem were strongly associated with male sex and lower age. This is in line with previous findings of an enduring sex gap in heavy drinking and alcohol use disorders [2, 17, 24, 38]. Men were less likely to drink heavily if they lived with a spouse or partner, whereas this association was not found in women. The fact that women drink heavily to a lesser extent than men has been suggested to be an expression of their role as controllers of the more risky drinking behaviours of men [41]. Although we found a considerably higher prevalence of heavy episodic drinking among men compared to women, i.e., 57% and 23%, respectively, it has been argued that AUDIT-3 underestimates health-impacting HED prevalence among older women [19]. The criterion of ≥6 drinks is not sex-adjusted according to different sensitivities to the adverse effects of alcohol and may be too high to identify harmful binge drinking among women. Our finding is in accordance with another study among older adults using the same AUDIT-3 threshold [19]; however, our study included older participants.

### Factors associated with at-risk drinking

Our findings of a strong link between higher education levels and at-risk drinking in both sexes and with alcohol problems in women are in line with other recent studies that describe a strong correlation between a higher education level and unhealthy drinking, which is even stronger for women [21, 26, 40]. Existing research indicates a change in the current generation of older adults’ perception of what behaviours are considered acceptable, which may have affected alcohol habits, including among the healthy and highly educated elderly [6]. The suggested under-detection of alcohol problems may be related to the fact that clinicians do not suspect at-risk alcohol use in this privileged group of elderly individuals [13].

Loneliness is prevalent among older adults and has been considered a risk factor for harmful drinking [8, 26, 42]. It has, however, also been found that loneliness is associated with reduced frequency of alcohol use [43]. Others report that older people explain that alcohol drinking is linked to pleasant social gatherings and that increased social engagement is associated with increased drinking [44, 45]. Our findings support the latter; the assumption that having enough friends protects against harmful drinking could therefore not be confirmed by our study. Inconsistent findings across studies suggest that risky alcohol consumption is probably linked to loneliness in complex ways [26, 42–44, 46]. Divergent social and possibly gender-related norms for how to deal with loneliness and how alcohol is used in social settings across countries may explain some of the conflicting findings.

Our finding of a strong link between good or excellent health and at-risk drinking, and worse health status and abstention from alcohol among women, but not among men, was surprising. In a large longitudinal study from England, Holdsworth et al. found that drinking frequency later in life may be an indicator of health status rather than the cause of health status [47]. Our study suggests that alcohol consumption is an indicator of health status in women, but possibly not in men, in Norway. It is well-known that former drinkers often stop consuming alcohol when their health status worsens, which is known as the “sick quitters” effect [47]. Our findings indicate that this effect applies especially to older women, supporting findings of gender differences regarding risky health behaviours [41].

Our study did not find an association between mental distress and at-risk drinking as measured with AUDIT-C or AUDIT-3. However, a total score on AUDIT items 4-10 ≥1, indicating some sort of alcohol problem, was strongly associated with mental distress in both men and women. Despite the fact that a causal linkage between excessive alcohol use and depression among the general adult population has been found [48], this is not a consistent finding among the elderly [21, 49–51].

Our findings may indicate that some older adults with mental distress self-medicate with alcohol and consume more alcohol, while others, especially women, stop drinking (S. Table 1).

It has been found that the proportion of older adults who combine alcohol and medication, such as opioids and benzodiazepines, has increased [5] and that men combine sedative hypnotics with alcohol more often than women [52]. In our study, the use of sleeping pills was strongly associated with both exceeding at-risk drinking thresholds and experiencing some sort of alcohol problem in men, while it was only associated with experiencing some sort of alcohol problem in women. The finding of a higher probability of using sleeping pills when also experiencing alcohol problems is concerning, as concomitant use of alcohol and sedative hypnotics can exacerbate CNS depression, cause drowsiness and increase the risk of falls and confusion [9, 52].

The adequacy of using a lower consumption threshold for older adults, and even sex-specific thresholds, has been subject to some debate [16, 19]. Bush et al., who validated the abbreviated version of the Alcohol Use Disorders Identification Test (AUDIT-C), recommended a limit of ≥3 to indicate at-risk alcohol consumption [53]. Since then, this cut-off limit, which was validated in a cohort prone to alcohol abuse, has been criticized [54]. Poor sensitivity of a measure results in overestimation of prevalence; thus, increasing the standard AUDIT-C threshold can enable a more sensitive and specific screen. An AUDIT-C threshold of ≥5 for at-risk drinking in the general population samples has been suggested [54], with a threshold of ≥4 in older adults [55]. However, due to naturally lower levels of body water in women than in men and sex differences in alcohol metabolism, women are more susceptible to adverse effects of alcohol [56]. Even if there is a risk of overestimation of prevalence, it has therefore been argued that utilizing both sex- and older adult-specific thresholds more validly identifies at-risk drinkers [19]. Moreover, there is a strong correlation between an AUDIT-C score ≥3 for older women and ≥4 for older men and exceeding the alcohol consumption limits recommended by the NIAAA [57].

There is currently little knowledge about whether older adults who exceed different defined at-risk drinking limits will develop alcohol use disorders in the future or whether at-risk drinking is better tolerated in subgroups of the elderly. Nevertheless, potentially harmful drinking in this fast-growing segment of the population is widespread, and this requires increased attention from health care providers.

#### Clinical implications

The findings of this study may be particularly important for clinicians to help identify older adults who may be at higher risk of physical illnesses, injuries or other health-related consequences due to risky alcohol use. At-risk drinkers fit into the perception of “successful aging”, with higher levels of education, better health and a larger degree of social satisfaction than low-risk drinkers, and this is especially true for women. Additionally, not having enough social support, living alone, mental distress, and consuming sleeping pills may also indicate alcohol problems in older adults.

#### Limitations

The attendance rate was lower in the oldest age groups and may therefore be less representative of the general population. Although our findings among the oldest have reduced power, we decided not to exclude this age group, as few studies that have included the oldest age group (aged 80 years and older) have been conducted. Caution must therefore be exercised in interpreting these results.

Underreporting in studies based on self-reporting questionnaires, as in our study, is often considered a problem [58]. However, our findings of such a high proportion of older adults reporting to exceed suggested older-specific AUDIT thresholds, can indicate more liberal attitudes towards alcohol use in old age, as reported by others [6]. Reduced discomfort by reporting alcohol consumption correctly can imply that underreporting may be a minor problem among the new cohort of older adults as compared to older cohorts [12].

As this study is cross-sectional, it is not possible to predict whether the younger cohorts will reduce their alcohol consumption as they age in the same way as the older cohorts in this study. There is international evidence that older adults in the current baby boomer generation do not decrease drinking while aging [59, 60], including in Norway [11].

The cross-sectional nature of this study means that the interpretation of correlated findings is challenging. Prospective longitudinal studies are therefore essential to further broaden the understanding of the directions of relationships between risk factors and risk drinking.

Alcohol misuse, abstaining from alcohol (“sick quitter syndrome”), and mental distress are moderately associated with non-participation in population surveys [61, 62]. The underrepresentation of people with high alcohol consumption, abstainers and people with poor mental health should be taken into consideration when interpreting results from population-based health surveys. Furthermore, if the deleted cases due to missing values were not missing at random, listwise deletion may have caused some biases in the estimates.

Polypharmacy and comorbidity are major concerns in combination with elevated alcohol consumption [9]. We did not have access to data on self-reported medications other than sleeping pills, which implies a limitation in the interpretation of the results.

The Tromsø Study is based in the seventh largest Norwegian city, with relatively few immigrants, and it is limited with regard to ethnic diversity. The generalizability of the results may therefore be limited to Caucasian populations that are similar to older adults of Norwegian descent. Furthermore, people living in urban areas drink more than those in rural areas [11]. Since our sample does not include rural-living older adults, the generalizability in prevalence rates of alcohol consumption may be restricted to urban-living older adults.

## Conclusions

Alcohol consumption is very prevalent among older adults in Norway, especially among highly educated adults. Furthermore, almost half of current drinkers exceeded sex- and older adult-specific thresholds for at-risk drinking among both women and men. At-risk drinking was strongly associated with very good health, living with a spouse or partner, and having adequate social support among women, while it was only associated with the use of sleeping pills among men. In both sexes, experiencing some sort of alcohol problem was associated with mental distress and the use of sleeping pills. Although the prevalence of at-risk drinking among those aged 60 years and older is high, it is not necessarily connected to alcohol-related harm. However, increased attention is required from health care professionals to detect and intervene in those at risk of health-related consequences due to excessive alcohol use. Future research should longitudinally investigate the health-related consequences of different patterns of at-risk drinking among the elderly.

## Supplementary Information

**Additional file 1:** **Table S1.** Factors associated with non-drinking, stratified by sex: Participants ≥60 years (*n *= 8,616) in the Tromsø survey (2015-16)

## Data Availability

The legal restriction on data availability are set by the Tromsø Study Data and Publication Committee in order to control for data sharing, including publication of datasets with the potential of reverse identification of de-identified sensitive participant information. We have received administrative permission to access and use the data that support the findings of this study. A detailed overview of the data collection process, including links to the main questionnaires, can be found on the website of the Tromsø Study (https://uit.no/research/tromsostudy). We do not have permission to share the data, but readers may contact Professor Sameline Grimsgaard, sameline.grimsgaard@uit.no to request the data or receive a confirmation that data will be available upon reasonable request to researchers.

## Abbreviations

CI: Confidence interval
HED: Heavy episodic drinking. Drinking ≥6 units (≥72 grams of ethanol) in one sitting
OR: Odds ratio
SRH: Self-reported health status

## Glossary

**AUDIT**: Alcohol Use Disorders Identification Test. The AUDIT is a ten-item alcohol screen that can help identify persons who are at-risk drinkers. Items 4-10 consist of questions about the negative consequences of alcohol consumption (Problems).
**AUDIT-C**: Alcohol Use Disorders Identification Test-Consumption. The AUDIT-C is recommended for identifying at-risk drinking prevalence in older adults, consisting of the first three items from the AUDIT regarding consumption.
**AUDIT-3**: Consists of the third item of AUDIT-C and serves as an initial screen to identify binge drinkers.
**At-risk Drinking**: Refers to the consumption of alcohol, on any single occasion or on average during one week, or exceeding suggested older-specific AUDIT thresholds, that is considered risky to one's health. Specific definitions vary across the literature because there are a number of methodological and conceptual challenges [20].
**Drinking Recommendations**: Recommendations that help people drink safely by suggesting levels of consumption that have been shown to be low-risk for injury or harm. They are commonly based on a “typical” person.
**HSCL-10**: The Hopkins Symptom Check List-10. The suggested cut-off limit of HSCL-10 indicating mental distress is ≥1.85.
**NIAAA**: The US National Institute on Alcohol and Alcoholism is the lead federal agency for research on alcohol and health and the largest funder of alcohol research in the world (https://www.niaaa.nih.gov/about-niaaa).

## References

[1] Geels LM (2013). Increases in alcohol consumption in women and elderly groups: evidence from an epidemiological study. BMC Public Health.

[2] Gell L, Meier PS, Goyder E (2015). Alcohol consumption among the over 50s: international comparisons. Alcohol Alcohol.

[3] Crome I, Dar K, Janikiewicz S, Rao T, Tarbuck A. Our invisible addicts. First Report of the Older Persons’ Substance Misuse Working Group of the Royal College of Psychiatrists. London: The Royal College of Psychiatrists; 2011. Available from https://www.rcpsych.ac.uk/docs/default-source/improving-care/better-mhpolicy/college-reports/college-report-cr211.pdf?sfvrsn=820fe4bc_2. Accessed 20 June 2021.

[4] Barry KL, Blow FC (2016). Drinking Over the Lifespan: Focus on Older Adults. Alcohol Res.

[5] Wang Y-P, Andrade LH (2013). Epidemiology of alcohol and drug use in the elderly. Curr Opin Psychiatry.

[6] Bareham BK (2019). Drinking in later life: a systematic review and thematic synthesis of qualitative studies exploring older people’s perceptions and experiences. Age Ageing.

[7] Rehm J (2017). The relationship between different dimensions of alcohol use and the burden of disease-an update. Addiction.

[8] Kuerbis A (2020). Substance Use among Older Adults: An Update on Prevalence, Etiology, Assessment, and Intervention. Gerontology.

[9] Moore AA, Whiteman EJ, Ward KT (2007). Risks of combined alcohol/medication use in older adults. Am J Geriatr Pharmacother.

[10] World Health Organization. Global status report on alcohol and health 2018. World Health Organization; 2019. Available from https://books.google.no/books?%20hl=en&lr=&id=qnOyDwAAQBAJ&oi=fnd&pg=PR7&dq=global+status+report+on+alcohol+2018&ots=a1oqRBvhhn&sig=x9JdmI9tIGFKnAaFPAeoA4ZeoWU&redir_esc=y#v=onepage&q=global%20status. Accessed 20 Jan 2022.

[11] Bye EK, Moan IS (2020). Trends in older adults’ alcohol use in Norway 1985–2019. Nordic Studies on Alcohol and Drugs.

[12] Stelander LT (2021). The changing alcohol drinking patterns among older adults show that women are closing the gender gap in more frequent drinking: the Tromsø study, 1994–2016. Subst Abuse Treat Prev Policy.

[13] Caputo F (2012). Alcohol use disorders in the elderly: a brief overview from epidemiology to treatment options. Exp Gerontol.

[14] Barnes AJ (2010). Prevalence and correlates of at-risk drinking among older adults: the project SHARE study. J Gen Intern Med.

[15] Hallgren M, Högberg P, Andréasson S (2009). Alcohol consumption among elderly European Union citizens, in Health effects, consumption trends and related issues.

[16] Crome I (2012). Alcohol limits in older people. Addiction.

[17] Blazer DG, Wu LT (2009). The epidemiology of at-risk and binge drinking among middle-aged and elderly community adults: National Survey on Drug Use and Health. Am J Psychiatry.

[18] NIAAA. Drinking Levels Defined. 2021 [cited 2021 21 December ]; Available from: https://www.niaaa.nih.gov/alcohol-health/overview-alcohol-consumption/moderate-binge-drinking.

[19] Towers A (2011). Estimating older hazardous and binge drinking prevalence using AUDIT-C and AUDIT-3 thresholds specific to older adults. Drug and Alcohol Dependence.

[20] Gilson KM, Bryant C, Judd F (2014). Exploring risky drinking and knowledge of safe drinking guidelines in older adults. Subst Use Misuse.

[21] Bosque-Prous M (2017). Hazardous drinking in people aged 50 years or older: a cross-sectional picture of Europe, 2011-2013. Int J Geriatr Psychiatry.

[22] Moore AA, Kuerbis A, Sacco P, Chen GI, Garcia MB. Screening and assessment of unhealthy alcohol use in older adults. In Alcohol and Aging. Cham: Springer; 2016. pp. 169-180

[23] Saunders JB (1993). Development of the Alcohol Use Disorders Identification Test (AUDIT): WHO Collaborative Project on Early Detection of Persons with Harmful Alcohol Consumption--II. Addiction.

[24] Blazer DG, Wu LT (2011). The epidemiology of alcohol use disorders and subthreshold dependence in a middle-aged and elderly community sample. Am J Geriatr Psychiatry.

[25] Mejldal A (2020). Twenty Years Socioeconomic Trajectories in Older Adults with Varying Alcohol Use: A Register-Based Cohort Study. Alcohol Alcohol.

[26] Iparraguirre J (2015). Socioeconomic determinants of risk of harmful alcohol drinking among people aged 50 or over in England. BMJ Open.

[27] Behrendt S (2020). Research is needed to understand substance use disorders in old adulthood. Addiction.

[28] Jacobsen BK (2012). Cohort profile: the Tromso Study. Int J Epidemiol.

[29] Idler EL, Benyamini Y (1997). Self-rated health and mortality: a review of twenty-seven community studies. J Health Soc Behav.

[30] Jylhä M (2009). What is self-rated health and why does it predict mortality? Towards a unified conceptual model. Soc Sci Med.

[31] Ganna A, Ingelsson E (2015). 5 year mortality predictors in 498,103 UK Biobank participants: a prospective population-based study. Lancet.

[32] Derogatis LR (1974). The Hopkins Symptom Checklist (HSCL): a self-report symptom inventory. Behav Sci.

[33] Schmalbach B (2021). Psychometric Properties of Two Brief Versions of the Hopkins Symptom Checklist: HSCL-5 and HSCL-10. Assessment.

[34] Søgaard AJ (2003). A comparison of the CONOR Mental Health Index to the HSCL-10 and HADS. Norsk epidemiologi.

[35] Tevik K (2017). Use of alcohol and drugs with addiction potential among older women and men in a population-based study. The Nord-Trøndelag Health Study 2006-2008 (HUNT3). PLoS One.

[36] Selin KH (2006). Alcohol Use Disorder Identification Test (AUDIT): what does it screen? Performance of the AUDIT against four different criteria in a Swedish population sample. Subst Use Misuse.

[37] Keyes KM, Li G, Hasin DS (2011). Birth cohort effects and gender differences in alcohol epidemiology: a review and synthesis. Alcohol Clin Exp Res.

[38] Wilsnack RW (2009). Gender and alcohol consumption: patterns from the multinational GENACIS project. Addiction.

[39] Bratberg GH (2016). Gender differences and gender convergence in alcohol use over the past three decades (1984-2008), The HUNT Study, Norway. BMC Public Health.

[40] Rossow I, Træen B (2020). Alcohol use among older adults: A comparative study across four European countries. Nordic Studies on Alcohol and Drugs.

[41] Holmila M, Raitasalo K (2005). Gender differences in drinking: why do they still exist?. Addiction.

[42] Immonen S, Valvanne J, Pitkälä KH (2011). Older adults’ own reasoning for their alcohol consumption. Int J Geriatr Psychiatry.

[43] Canham SL (2016). Association of Alcohol Use and Loneliness Frequency Among Middle-Aged and Older Adult Drinkers. J Aging Health.

[44] Dare J (2014). Social engagement, setting and alcohol use among a sample of older Australians. Health Soc Care Community.

[45] Nicholson D (2017). Alcohol and healthy ageing: a challenge for alcohol policy. Public Health.

[46] Hajek A (2017). Correlates of alcohol consumption among Germans in the second half of life. Results of a population-based observational study. BMC Geriatr.

[47] Holdsworth C (2016). Is regular drinking in later life an indicator of good health? Evidence from the English Longitudinal Study of Ageing. J Epidemiol Community Health.

[48] Boden JM, Fergusson DM (2011). Alcohol and depression. Addiction.

[49] Gea A (2013). Alcohol intake, wine consumption and the development of depression: the PREDIMED study. BMC Med.

[50] Bellos S (2016). Longitudinal association between different levels of alcohol consumption and a new onset of depression and generalized anxiety disorder: Results from an international study in primary care. Psychiatry Res.

[51] Bryant AN, Kim G (2013). The relation between frequency of binge drinking and psychological distress among older adult drinkers. J Aging Health.

[52] Ilomaki J (2013). Prevalence of concomitant use of alcohol and sedative-hypnotic drugs in middle and older aged persons: a systematic review. Ann Pharmacother.

[53] Bush K (1998). The AUDIT alcohol consumption questions (AUDIT-C): an effective brief screening test for problem drinking. Ambulatory Care Quality Improvement Project (ACQUIP). Alcohol Use Disorders Identification Test. Arch Intern Med.

[54] Rumpf HJ (2002). Screening for alcohol use disorders and at-risk drinking in the general population: psychometric performance of three questionnaires. Alcohol Alcohol.

[55] Dawson DA (2005). Effectiveness of the derived Alcohol Use Disorders Identification Test (AUDIT-C) in screening for alcohol use disorders and risk drinking in the US general population. Alcohol Clin Exp Res.

[56] Bradley KA (1998). Medical risks for women who drink alcohol. J Gen Intern Med.

[57] Rubinsky AD (2013). AUDIT-C scores as a scaled marker of mean daily drinking, alcohol use disorder severity, and probability of alcohol dependence in a U.S. general population sample of drinkers. Alcohol Clin Exp Res.

[58] Gmel G, Rehm J (2004). Measuring Alcohol Consumption. Contemporary Drug Problems.

[59] Ilomäki J (2010). Changes in alcohol consumption and drinking patterns during 11 years of follow-up among ageing men: the FinDrink study. Eur J Public Health.

[60] Veerbeek MA (2019). Differences in alcohol use between younger and older people: Results from a general population study. Drug Alcohol Depend.

[61] Zhao J, Stockwell TIM, Macdonald S (2009). Non–response bias in alcohol and drug population surveys. Drug and Alcohol Review.

[62] Lundberg I (2005). Determinants of non-participation, and the effects of non-participation on potential cause-effect relationships, in the PART study on mental disorders. Soc Psychiatry Psychiatr Epidemiol.

