# Supplementary material for: Sex differences in at-risk drinking and associated factors–a cross-sectional study of 8,616 community-dwelling adults 60 years and older: the Tromsø study, 2015-16
Source: BMC Geriatr. 2022 Mar 1;22:170. doi: 10.1186/s12877-022-02842-w (PMC8886794; doi:10.1186/s12877-022-02842-w)
Supplement: Supplementary file 1 — Additional file 1: Table S1. Factors associated with non-drinking, stratified by sex: Participants ≥60 years (n = 8,616) in the Tromsø survey (2015-16) [file 12877_2022_2842_MOESM1_ESM.docx]

### S. Table 1 Factors associated with non-drinking, stratified by sex: Participants ≥60 years (n=8,616) in the Tromsø survey (2015-16)

|  |  | **Men**  Non-drinker (n=305) vs. current drinker (n=3,830) | |  | **Women**  Non-drinker (n=639) vs. current drinker (n=3,752) | |
| --- | --- | --- | --- | --- | --- | --- |
| Predictor |  | Adjusted OR | 95% CI |  | Adjusted OR | 95% CI |
| Age (ref group: 60-69) |  | 1.00 | ref |  | 1.00 | ref |
| 70–79 |  | 1.75** | 1.34-2.29 |  | 2.15** | 1.77-2.61 |
| ≥80 |  | 3.77** | 2.67-5.34 |  | 3.58** | 2.74-4.67 |
| Education (ref group: Elementary school) |  | 1.00 | ref |  | 1.00 | ref |
| High school |  | 0.73* | 0.55-0.98 |  | 0.56** | 0.44-0.70 |
| College/university |  | 0.50** | 0.37-0.67 |  | 0.46** | 0.36-0.73 |
| Living with a spouse or partner (vs. living alone) |  | 0.65** | 0.49-0.87 |  | 0.72** | 0.59-0.87 |
| Enough social support (vs. not) |  | 0.73* | 0.53-0.99 |  | 0.63** | 0.49-0.81 |
| Self‐reported health status (ref group: Bad or very bad) |  | 1.00 | ref |  | 1.00 | ref |
| Neither good nor bad |  | 0.94 | 0.57-1.54 |  | 0.44** | 0.32-0.61 |
| Good |  | 0.58* | 0.36-0.96 |  | 0.29** | 0.21-0.40 |
| Excellent |  | 0.78 | 0.42-1.44 |  | 0.23** | 0.14-0.36 |
| Mental distress (vs. no mental distress) |  | 1.66 | 0.91-3.02 |  | 1.82** | 1.32-2.51 |
| Have used sleeping pills during last 4 weeks (vs. no use last 4 weeks) |  | 1.20 | 0.80-1.81 |  | 1.37** | 1.09-1.72 |

OR, Odds Ratio; CI, Confidence Interval

†Adjusted for age (continuous) and educational level

*p≤0.05 **p<0.01
